# Emergence of Extensively Drug-Resistant Shigellosis — United States, 2011–2023

**DOI:** 10.15585/mmwr.mm7513a1

**Published:** 2026-04-09

**Authors:** Naeemah Logan, Meseret G. Birhane, Sharla L. McDonald, Jemma Alarcón, Salette Amador, Madhu Anand, Laura H. Bachmann, Kevin Bernard, Lindsay S. Black, Angela N. Chen, Laura A. Cooley, Esther D. Fortes, Marisa Gerard, Rachel H. Jervis, Akiko C. Kimura, Katherine Lamba, Julia Latash, Linlin Li, Grace Pederson, Carley Perez Kauffman, Jared L. Reynolds, Richard Santiago, Ulrike Siemetzki-Kapoor, Sandra C. Smole, Kaitlin A. Tagg, Johanna Vostok, Hattie E. Webb, Louise K. Francois Watkins, XDR Shigella Working Group, XDR Shigella Working Group, Shana M. Altman, Kyli Beaulieu, Oliver M. Beaumont, Raychel Berkheimer, Brianna Loeck, Sean C. Buuck, Lisandra Clarke, Matthew Collins, Irina Cody, Stephen Combes, MaryBeth DeMarco, Sarah Feeney, Michael Gosciminski, Samir Hanna, Noël V. Hatley, Peter C. Iwen, Jennifer A. Khoury, Lauren Kleamenakis, Julia Maeda, Jack Marr, Naiyma Martin, Nkuchia M. M'ikanatha, Nancy Persson, Craig Rothfuss, Carrell T. Rush, Sameera Sayeed, Sarah Shrum Davis, Shamika Smith, Laurie K. Stewart, Quoc Phung Than, Rosalie T. Trevejo, Christina L. Turner, Elena Washington, Sophia Wozny, Mary Whisnant, Michelle Yang

**Affiliations:** ^1^Division of Foodborne, Waterborne, and Environmental Diseases, National Center for Emerging and Zoonotic Infectious Diseases, CDC; ^2^Los Angeles County Department of Public Health, Los Angeles, California; ^3^New York State Department of Health; ^4^Division of STD Prevention, National Center for HIV, Viral Hepatitis, STD, and Tuberculosis Prevention, CDC; ^5^New York City Department of Health and Mental Hygiene, New York, New York; ^6^Florida Department of Health; ^7^Massachusetts Department of Public Health; ^8^Colorado Department of Public Health and Environment; ^9^California Department of Public Health; ^10^Great Hill Solutions, A Seneca Nation Group Company, Chantilly, Virginia.; Illinois Department of Public Health; Iowa Department of Health and Human Services; New Hampshire Department of Health and Human Services; Louisiana Department of Health; Nebraska Department of Health and Human Services; Minnesota Department of Health; One Health Delaware Program; Indiana Department of Health; Texas Department of State Health Services; Maine Center for Disease Control and Prevention; Virginia Department of Health; Indiana Department of Health; Rhode Island Department of Health; Tennessee Department of Health; Washington State Department of Health; Nebraska Public Health Laboratory; Kentucky Department for Public Health; Louisiana Department of Health; New Hampshire Department of Health and Human Services; Tennessee Department of Public Health; New Mexico Department of Health; Pennsylvania Department of Health; Rhode Island Department of Health; Maine Center for Disease Control and Prevention; MCD Global Health; Kentucky Department for Public Health; Pennsylvania Department of Health; New Mexico Department of Health; Chicago Department of Public Health; Washington State Department of Health; Texas Department of State Health Services; Oregon Health Authority; Connecticut Department of Public Health; Connecticut Department of Public Health; Maryland Department of Health; Arkansas Department of Health; Arkansas Department of Health.

**Summary**

**What is already known about this topic?**

U.S. cases of extensively drug-resistant (XDR) *Shigella* infections are increasing; no Food and Drug Administration–approved oral treatment is available. *Shigella* bacteria are easily transmissible and resistance genes can spread to other enteric bacteria, making XDR *Shigella* a public health threat. U.S. trends have not been fully described.

**What is added by this report?**

Among nearly 17,000 *Shigella* species isolates with resistance data, XDR isolates increased from 0% in 2011 to 8.5% in 2023. Whereas earlier U.S. outbreaks involved drug-susceptible strains and primarily affected children, national surveillance data indicate that most XDR cases occurred among adult men. Approximately one third of patients were hospitalized.

**What are the implications for public health practice?**

The high transmission potential of XDR *Shigella* strains highlights the importance of susceptibility testing and timely reporting of this nationally notifiable disease for prevention.

## Abstract

Shigellosis is a nationally notifiable diarrheal illness caused by gram-negative bacteria. *Shigella* infection is spread through fecal-oral transmission and sexual contact. Although most infections are self-limited, antibiotics are indicated for severe illness or to reduce transmission in settings with high risk for spread. Since 2015, a growing proportion of cases has been caused by extensively drug-resistant (XDR) *Shigella* species, defined as being resistant to ampicillin, azithromycin, ceftriaxone, ciprofloxacin, and trimethoprim-sulfamethoxazole. No Food and Drug Administration–approved oral antimicrobial agents are available to treat these XDR infections. To describe U.S. trends and epidemiologic characteristics of XDR shigellosis, CDC analyzed *Shigella* isolates submitted to PulseNet, CDC’s molecular surveillance network for enteric pathogens, during January 1, 2011–October 20, 2023; antimicrobial resistance was characterized using whole-genome sequencing data and antimicrobial susceptibility testing. Among 16,788 isolates with resistance data during this period, 510 (3.0%) were XDR. The percentage of XDR isolates increased from 0% during 2011–2015 to 8.5% in 2023. Species information was available for 505 (99%) of 510 XDR isolates; among those, 333 (65.9%) were *Shigella sonnei* and 172 (34.1%) were *Shigella flexneri.* Among patients with XDR shigellosis, the median patient age was 41 years (IQR = 31–54 years) and 86.2% were men. Among patients with available travel history, 76.2% (173 of 227) reported no recent domestic travel and 82.4% (169 of 205) reported no recent international travel. Among 116 persons with available HIV status, 54 (46.6%) reported HIV co-infection. Strengthened surveillance, timely reporting, and targeted prevention strategies are needed to limit transmission of XDR *Shigella* strains.

## Introduction

*Shigella* species are highly infectious gram-negative bacteria that cause acute diarrheal illness and spread easily from person to person through fecal-oral transmission or sexual contact, or through contaminated food, water, or fomites. Infection can occur with as few as 10 organisms. Most infections resolve without requiring treatment with antimicrobial agents; however, antibiotics are indicated for severe illness or to reduce transmission in high-risk settings (*1*). In the United States, shigellosis is a nationally notifiable disease. Extensively drug-resistant (XDR) *Shigella* (defined as *Shigella* species isolates that are resistant to ampicillin, azithromycin, ceftriaxone, ciprofloxacin, and trimethoprim-sulfamethoxazole) is a public health concern because no Food and Drug Administration–approved oral antimicrobial agents are available, alternative oral treatment options are limited, and resistance genes can spread to other enteric bacteria. The proportion of XDR *Shigella* among all isolates has increased, and national trends and epidemiologic characteristics have not been fully described. To address this gap, CDC examined antimicrobial resistance patterns of *Shigella* isolates that were submitted to PulseNet, CDC’s molecular surveillance network for foodborne diseases, during January 1, 2011 (when azithromycin was added to CDC’s National Antimicrobial Resistance Monitoring System [NARMS] panel) through October 20, 2023. This report describes temporal trends in the proportion of XDR *Shigella* isolates and characterizes demographic, clinical, and epidemiologic features of patients with XDR shigellosis to help guide the development and implementation of prevention strategies to limit transmission.

## Methods

### Data Source

State and local public health laboratories submit *Shigella* isolates to PulseNet for routine surveillance and outbreak detection; submissions are determined by jurisdictional protocols and laboratory capacity, rather than by patient-level clinical or demographic factors. During January 1, 2011–October 20, 2023, a total of 16,788 *Shigella* isolates submitted to PulseNet had resistance data from whole-genome sequencing (WGS), antimicrobial susceptibility testing (AST), or both. WGS and AST represent different but highly concordant methods used to track antimicrobial resistance in enteric bacteria for surveillance purposes (*2*). Of the 16,788 isolates, 1,510 had AST data, including 1,454 with both AST and WGS; 56 had AST only, and 15,278 had WGS only. Use of WGS increased over the study period as sequencing capacity expanded nationally. Trends were interpreted using counts and proportions. Some epidemiologic variables, including sexual exposure, housing status, and sexually transmitted infection (STI) co-infection, were not routinely collected for *Shigella* cases and were therefore not analyzed.

### Antimicrobial Resistance Testing

Antimicrobial resistance was characterized by NARMS using AST or WGS. AST used broth microdilution with Sensititer panels (Thermo Fisher Scientific; panel types varied) per manufacturer’s instructions and Clinical and Laboratory Standards Institute (CLSI) guidelines. Results were interpreted using CLSI M100 (35th edition; 2025) clinical breakpoints where available. Resistance determinants (genetic markers that predict phenotypic antimicrobial resistance) were identified in WGS data using ResFinder and PointFinder databases (*3*). Isolates were classified as XDR if 1) they were resistant by AST to ampicillin, azithromycin, ceftriaxone, ciprofloxacin, and trimethoprim-sulfamethoxazole or 2) WGS identified resistance determinants for each of these agents (at least two for ciprofloxacin). In addition to the five antibiotics used to define XDR, isolates were tested for resistance to other agents on NARMS panels, including chloramphenicol and meropenem. Resistance determinants for fosfomycin were identified by WGS. Epidemiologic variables requested for XDR cases included age, sex, race, ethnicity, HIV status, and reported domestic and international travel history. Data were reviewed in the System for Enteric Disease Response, Investigation, and Coordination, CDC’s secure platform for outbreak investigations and coordination. Frequencies and percentages were calculated in Stata (version 15.1; StataCorp) and stratified by U.S. Census Bureau region. This activity was reviewed by CDC, deemed not research, and was conducted consistent with applicable federal law and CDC policy.*

## Results

### Characteristics of XDR *Shigella* Isolates

During January 2011–October 2023, a total of 510 XDR *Shigella* isolates were identified; species information was available for 505 (99%) of these, and the first XDR isolates were identified in 2016. These XDR isolates account for 3.0% of 16,788 *Shigella* isolates with resistance information. The percentage classified as XDR increased from 0% during 2011–2015 to 8.5% in 2023 (Figure), and 167 (32.7%) XDR isolates were also found to be resistant to chloramphenicol; no resistance to meropenem or fosfomycin was identified.

**FIGURE:**
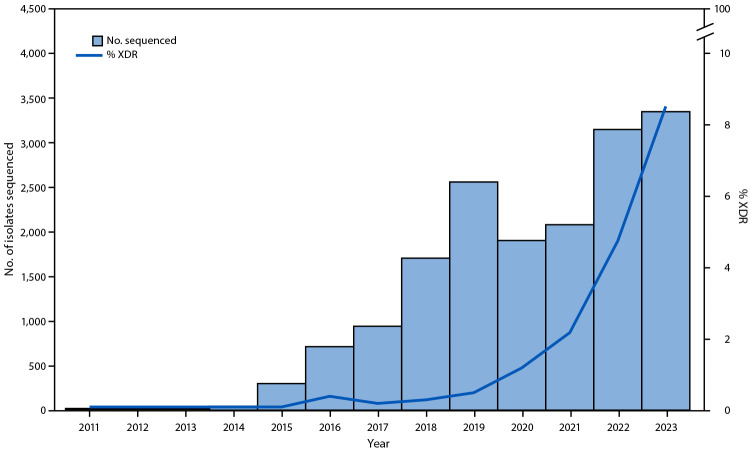
Number of sequenced *Shigella* isolates submitted to PulseNet and percentage that were extensively drug resistant*^,^^†^ — United States, 2011–2023 **Abbreviations:** AST = antimicrobial susceptibility testing; WGS = whole-genome sequencing; XDR = extensively drug resistant. * XDR *Shigella* infections are resistant to ampicillin, azithromycin, ceftriaxone, ciprofloxacin, and trimethoprim-sulfamethoxazole, based on AST, WGS, or both. ^†^ Of 16,788 isolates with resistance data, 15,278 had WGS only, 56 had AST only, and 1,454 had both WGS and AST data. Percent XDR was calculated among all isolates with resistance data (16,788).

### Characteristics of Patients with XDR Shigellosis

The median age of persons with XDR shigellosis was 41 years (IQR = 31–54 years) (Table 1). Among 492 persons with available data on sex and age, 473 (96.1%) were aged ≥18 years, including 424 (86.2%) men and 49 (10.0%) women; 19 (3.9%) cases occurred in persons aged <18 years (Supplementary Table). During the analysis period, most XDR cases (415; 84.3%) occurred during 2022–2023. Among 388 persons with available race data, 292 (75.3%) identified as White, 46 (11.9%) as Black or African American, 20 (5.2%) as Asian, three (0.8%) as American Indian or Alaska Native, two (0.5%) as Native Hawaiian or Pacific Islander, six (1.5%) as multiracial, and 19 (4.9%) as other (Table 1). Among 270 persons with ethnicity data, 52 (19.3%) identified as Hispanic or Latino. Among 116 patients with HIV status available, 54 (46.6%) reported HIV co-infection. Among 258 patients for whom hospitalization data were available, 97 (37.6%) were hospitalized; information on duration of hospitalization was not available. No deaths were reported.

**TABLE 1:** Characteristics of persons with extensively drug-resistant shigellosis — United States, 2011–2023

Characteristic (no. with available data)	No. (%)
**Age group and sex (492)**	
≥18 yrs	473 (96.1)
Female	49 (10.0)
Male	424 (86.2)
<18 yrs	19 (3.9)
Female	9 (1.8)
Male	10 (2.0)
Median age, yrs (IQR)	41 (31–54)
**Race and ethnicity (388)**	
American Indian or Alaska Native	3 (0.8)
Asian	20 (5.2)
Black or African American	46 (11.9)
Native Hawaiian or Pacific Islander	2 (0.5)
White	292 (75.3)
Multiracial	6 (1.5)
Other	19 (4.9)
Hispanic or Latino (270)	52 (19.3)
**HIV co-infection (116)**	54 (46.6)
**Hospitalization and death (258)**	
Hospitalization	97 (37.6)
Death	0 (—)
**Travel history**	
Domestic (227)	54 (23.8)
International (205)	36 (17.6)
**Specimen source (498)**	
Stool	483 (97.0)
Blood	9 (1.8)
Other*	6 (1.2)

* Colon (one), rectal swab (one), tissue (one), and other sources (three).

### Travel-Associated Cases

Information on domestic travel history was available for 227 persons, 54 (23.8%) of whom reported domestic travel and 173 (76.2%) who reported no domestic travel. Among 205 persons for whom information on international travel history was available, 36 (17.6%) reported international travel and 169 (82.4%) reported no international travel. Interpretation was limited by missing dates of travel relative to illness onset.

### Specimen Source, *Shigella* Species, and Geographic Distribution

Among 498 isolates with known specimen source, 483 (97.0%) were from stool and nine (1.8%) were from blood; the remaining six (1.2%) were from colon (one), rectal swab (one), tissue (one), and other sources (three). Among the 505 XDR isolates with species data, 65.9% were *Shigella sonnei* and 34.1% were *Shigella flexneri* (Table 2). By comparison, *S. flexneri* made up 18.5% of all sequenced *Shigella* isolates during the same period. XDR *Shigella* trends and species distribution were examined by U.S. Census Bureau region. The percentage of XDR cases caused by *S. flexneri* varied by location and time, peaking at 84.6% in the Northeast Region during 2021. In the West Region, the percentage reached 54.8% in 2023; in the Midwest Region, *S. flexneri* was first reported in 2023. The highest percentages of *S. flexneri* isolates among XDR cases were in Oregon (15 of 21; 71.4%), California (48 of 111; 43.2%), and Colorado (24 of 60; 40.0%). Some data fields, including sexual exposure, housing status, and STI co-infection, were incomplete and therefore not analyzed.

**TABLE 2:** Percentage of extensively drug-resistant Shigella* *flexneri* isolates among all XDR *Shigella* species isolates,^†^ by U.S. Census Bureau region^§^ and year — United States, 2011–2023^¶^

Year	Northeast		Midwest		South		West		Total	
	Total no. XDR*	No. (%) S. flexneri**	Total no. XDR*	No. (%) S. flexneri**	Total no. XDR*	No. (%) S. flexneri**	Total no. XDR*	No. (%) S. flexneri**	Total no. XDR*	No. (%) S. flexneri**
2016	1	0 (—)	0	0 (—)	0	0 (—)	1	0 (—)	**2**	**0 (—)**
2017	0	0 (—)	0	0 (—)	0	0 (—)	1	1 (100.0)	**1**	**1 (100.0)**
2018	0	0 (—)	0	0 (—)	3	0 (—)	0	0 (—)	**3**	**0 (—)**
2019	1	0 (—)	1	0 (—)	2	0 (—)	6	2 (33.3)	**10**	**2 (20.0)**
2020	10	0 (—)	2	0 (—)	0	0 (—)	9	2 (22.2)	**21**	**2 (9.5)**
2021	13	11 (84.6)	1	0 (—)	5	2 (40.0)	24	6 (25.0)	**43**	**19 (44.2)**
2022	26	15 (57.7)	11	0 (—)	22	9 (40.9)	86	33 (38.4)	**145**	**57 (39.3)**
2023	74	28 (37.8)	47	5 (10.6)	66	7 (10.6)	93	51 (54.8)	**280**	**91 (32.5)**
**Total**	**125**	**54 (43.2)**	**62**	**5 (8.1)**	**98**	**18 (18.4)**	**220**	**95 (43.2)**	**505**	**172 (34.1)**

**Abbreviation**: XDR = extensively drug resistant.

* XDR *Shigella* species isolates are defined as isolates that are resistant to ampicillin, azithromycin, ceftriaxone, ciprofloxacin, and trimethoprim-sulfamethoxazole.

^†^ Five of 510 XDR isolates were excluded because species data were missing.

^§^ New York City and Los Angeles County were classified within the Northeast and West U.S. Census Bureau regions, respectively.

^¶^ No XDR *Shigella* cases were reported during 2011–2015; the first XDR cases were identified in 2016. The study period begins in 2011 because azithromycin was added to the National Antimicrobial Resistance Monitoring System testing panel that year, and resistance to azithromycin is part of the XDR case definition.

** Among all XDR *Shigella* species isolates per region per year.

## Discussion

The percentage of *Shigella* isolates with resistance data that were XDR increased from 0% during 2011–2015 to 8.5% in 2023. Historically in the United States, shigellosis, a nationally notifiable disease, primarily affected children and was most often caused by drug-susceptible strains (*1*,*4*). In contrast, during 2016–2023, persons with XDR shigellosis for whom demographic data were available were predominantly non-Hispanic White men. Sexual exposure data were incomplete and not analyzed; however, previous studies identified sexual contact among men who have sex with men as an important *Shigella* transmission route (*5*). The emergence of XDR strains raises concerns for persons with immunocompromise, including those with HIV, for whom treatment options are limited and risk for severe illness is higher (*5*,*6*). XDR *Shigella* co-infection with other bacterial STIs has also been reported (*7*).

Species-specific factors were notable. *S. sonnei* has historically predominated in U.S. surveillance (*4*). Although *S. sonnei* still accounted for most XDR cases, the percentage of *S. flexneri* XDR isolates (34.1%) was almost twice that in overall U.S. *Shigella* surveillance (18.5%). This higher proportion of *S. flexneri* among XDR isolates might reflect network-related factors or regional antimicrobial pressures rather than intrinsic biologic differences between species (*8*). Published studies associate *S. flexneri* with more severe outcomes, including dysentery, hospitalization, and a higher case-fatality rate (*1*). The combination of XDR strains, possible higher virulence of *S. flexneri*, and risk among persons with immunocompromise warrants further study. Interpretation of reported travel histories was limited by missing data and lack of timing information.

Clinicians should rely on AST results from a clinical laboratory to guide therapy when possible. Treatment of XDR shigellosis remains challenging because no optimal therapy has been established. Chloramphenicol is not routinely recommended for shigellosis in the United States. Pivmecillinam, fosfomycin, and oral carbapenems (e.g., sulopenem) might be effective (*1*,*9*), but as of 2025, none was approved by the Food and Drug Administration (FDA) for shigellosis.

### Limitations

The findings in this report are subject to at least four limitations. First, surveillance likely underestimated XDR *Shigella* isolate incidence: not all isolates were sequenced or had AST, many specimens that were positive by culture-independent diagnostic tests were not cultured, underdiagnosis and incomplete reporting occurred, and submissions varied by jurisdiction over time. Second, AST results were not available for all isolates; however, given the high (>95%) concordance between AST and WGS, WGS is considered an acceptable method for surveillance (*2*). Third, WGS was limited before 2019, and state sequencing capacity varies, which might contribute to regional differences and observed increases in XDR detection. Finally, demographic, behavioral, and clinical data were often incomplete, surveillance capacity varied across jurisdictions, and travel history was missing for approximately one half of patients with XDR shigellosis.

### Implications for Public Health Practice

XDR *Shigella* infection is an emerging concern in the United States. Because no oral antimicrobial agents are FDA approved, prevention, early detection, AST-guided therapy, and timely reporting are important to protect populations at higher risk for XDR *Shigella* infection (*10*).

## Footnotes

* 45 C.F.R. part 46.102(l)(2), 21 C.F.R. part 56; 42 U.S.C. Sect. 241(d); 5 U.S.C. Sect. 552a; 44 U.S.C. Sect. 3501 et seq.
